# Strength Characteristics of Straw-Containing Cemented Tailings Backfill Under Different Strain Rates

**DOI:** 10.3390/ma18174193

**Published:** 2025-09-06

**Authors:** Zeyu Li, Xiuzhi Shi, Xin Chen, Jinzhong Zhang, Wenyang Wang, Xiaoyuan Li

**Affiliations:** 1 School of Resources and Safety Engineering, Central South University, Changsha 410083, China; lizeyu@csu.edu.cn (Z.L.);; 2 Sinosteel Maanshan General Institute of Mining Research Co., Ltd., Ma’anshan 243000, China; 3 School of Civil and Resources Engineering, University of Science and Technology Beijing, Beijing 100083, China; 4 Daye Nonferrous Mentals Group Holdings Co., Ltd., Huangshi 435000, China; 5 Fankou Lead-Zinc Mine, Shaoguan 512325, China; 6 Guangxi Zhongjin Lingnan Mining Co., Ltd., Laibin 545900, China

**Keywords:** rice straw fiber, cemented tailings backfill, unconfined compressive strength, dynamic increase factor

## Abstract

The frequent blasting in underground mines results in stress waves of different intensities, which is one of the main factors leading to backfill collapse. Improving the strength of backfill is an effective way to reduce the backfill damage. In this study, rice straw fiber and graded tailings were used as raw materials to prepare rice straw fiber-reinforced cemented tailings backfill (RSCTB). An orthogonal experimental design was employed to perform unconfined compressive strength (UCS) tests, diffusivity measurements, and Split Hopkinson Pressure Bar (SHPB) tests. The results showed that straw fibers slightly reduce slurry fluidity. The UCS of RSCTB at a specific mix ratio was more than 50% higher than that of cemented tailings backfill (CTB) without rice straw. The dynamic unconfined compressive strength (DUCS) of RSCTB increased linearly at different strain rates. The effect of rice straw fibers on the UCS and DUCS was much smaller than that of cement content and solid mass concentration. Excessively long and abundant straw fibers are not conducive to improving the long-term impact resistance of RSCTB.

## 1. Introduction

Cemented tailings backfilling mining is generally recognized as an environmentally friendly and safe mining method. Cemented tailings backfill (CTB) helps mitigate surface damage and environmental pollution associated with tailings storage facilities, as well as the risk of dam failure [1,2]. At the same time, it enhances local stability, prevents goaf collapse, and reduces rock strata subsidence [3,4,5]. To ensure that the backfill does not undergo significant collapse under stress waves generated by blasting, a substantial amount of cement is required to maintain adequate strength. Cement costs account for 50% to 75% of the filling slurry preparation process [6,7,8]. In pursuit of profits, technicians and researchers are striving to reduce costs while meeting safety standards.

Adding cheap and accessible materials to achieve the required strength is one of the methods. For the purpose of enhancing the strength, stiffness, and residual strength of CTB, researchers have attempted to incorporate low-cost fiber materials to improve its overall performance. Chen et al. [9,10] investigated the compressive, tensile, and microstructural properties of CTB reinforced with polypropylene fibers, and the results showed that polypropylene fibers can improve the stiffness and ductility of CTB, increase the residual strength of CTB after damage, and reduce the porosity of CTB. Cristelo et al. [11] found that under indirect tensile stress, the strength and strain of cement-stabilized sandy clay increased with the increase in polypropylene fiber content. Huang et al. [12] found that the average toughness index of CTB with added fiberglass increased in three-point bending experiments, and the fiberglass hindered crack extension. Juradin et al. [13] found that Spanish broom fibers can significantly improve the ductility of cementitious materials. It is generally accepted that the incorporation of fibers bridges internal microcracks and reduces material brittleness [14].

Compared with synthetic fibers such as polypropylene and glass fibers, rice straw (RS), as an agricultural byproduct, is more widely available, cost-effective, and environmentally friendly. Chen et al. [15] used straw to make cemented tailings fillers and found that the unconfined compressive strength of more than 85% of the CTBs and the modulus of elasticity of more than 74% of the CTBs increased more than twofold. Li et al. [16] found through Brazilian splitting experiments that, compared to conventional CTB, the tensile strength of rice straw fiber-reinforced cemented tailings backfill (RSCTB) increased by 115.38% to 300.00% on the 3rd day and 40.91% to 346.15% on the 7th day. Straw burning and in situ treatment cause a series of environmental problems, such as soot, oxynitride, sulfur dioxide pollution, and carbon dioxide emissions [17,18,19]. If straw fiber is used as an additive to enhance the performance of mine backfill, it will not only help to increase the utilization value of rice straw, but also help to alleviate these problems, which has great potential for application.

Unlike surface construction materials, backfill, once solidified, is subjected not only to long-term static loads but also to high strain rate loads caused by frequent blasting and seismic activities in underground mining [20]. Collapse of the backfill will reduce its limiting effect on rock deformation and will also exacerbate depletion of the ore during shoveling, increasing the burden on the beneficiation process and reducing productivity [21]. Dynamic strength should be considered when determining the strength of backfill [22,23]. It is feasible and economical to improve the strength of the filling body through straw fibers, but the lack of research on the dynamic compressive strength of RSCTB at different strain rates hinders its possible application in mines.

The Split Hopkinson Pressure Bar (SHPB) test has been widely used to test the high strain rate response of concrete [24,25], rocks [26,27], and ceramics [28,29], and likewise to test the impact resistance of backfill [30,31,32]. Tan et al. [31] found a quadratic relationship between dynamic peak stress and average stress rate by SHPB with different cement contents, and that an increase in cement content can increase the rate of increase in dynamic peak stress with strain rate. Cao et al. [33] conducted CTB dynamics experiments considering different loading rates and SHPB impact effects, and quantitatively investigated the relationship between the peak strength of the backfill and the dynamic properties. It is feasible to obtain the dynamic compressive properties of RSCTB by loading at different strain rates through SHPB, and it is of great significance to deeply understand how straw affects the dynamic compressive properties of RSCTB and improves its application in mining industry.

To fill the research gap in the high strain rate response of RSCTB and enhance the efficient utilization of agricultural waste, this study performed UCS and SHPB tests on RSCTB. The effects of RS fiber content, fiber length, and strain rate on the compressive behavior of RSCTB were examined. Furthermore, the study explored the microstructure of RSCTB, the effect of RS fibers on the flowability of the backfill slurry, and their roles in the macroscopic failure mechanisms.

## 2. Materials and Methods

### 2.1. Materials

**Figure 1:**
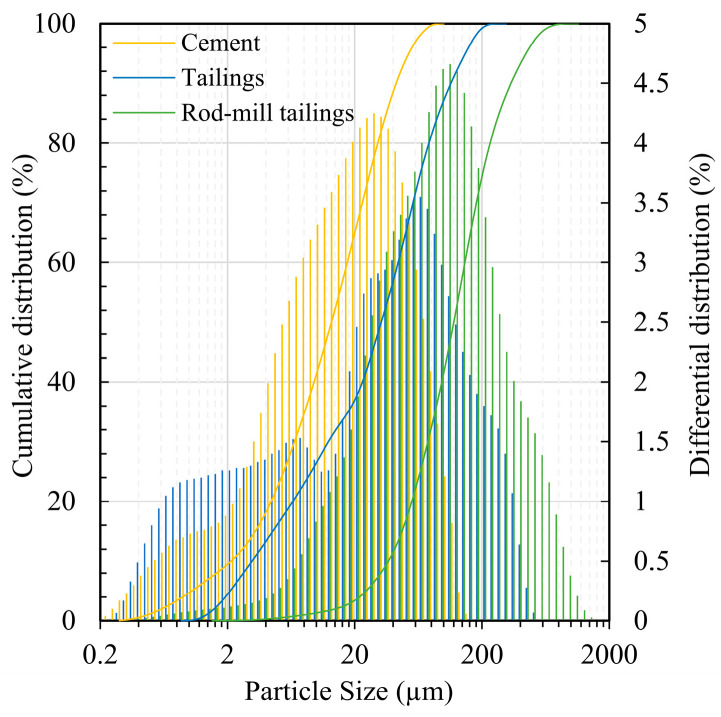
Particle size distribution of cement and tailings.

**Figure 2:**
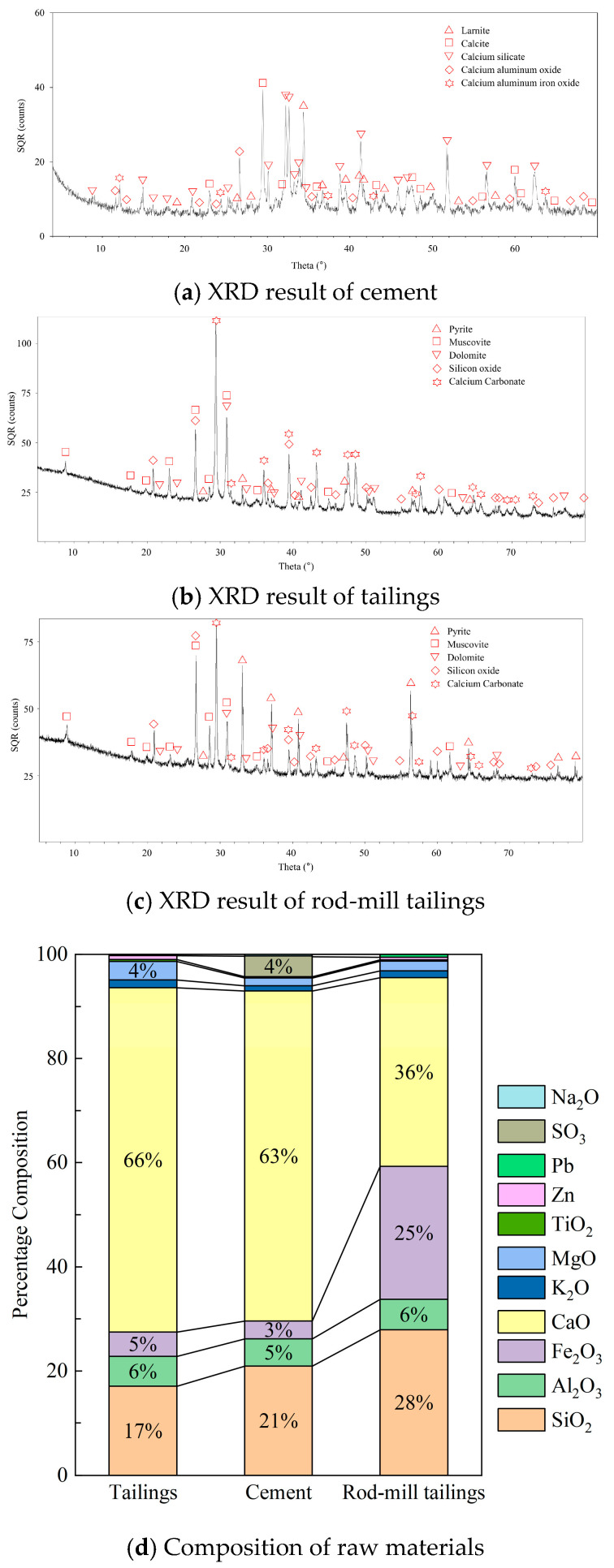
Mineralogical composition of cement, tailings, and rod-mill tailings.

The lead-zinc tailings for the preparation of CTB specimens in this experiment were from the Fankou lead-zinc mine in Shaoguan, China. Portland cement P.O 42.5R was used as the binder. Other raw materials were rod-mill tailings and tap water. The rod-mill tailings are the solid waste produced during the grinding process of the stone in rod mills. The particle sizes of tailings and cement were measured with an LS particle size analyzer (LS13320, Beckman, Brea, CA, USA), and the results are shown in Figure 1. The median particle size *D*_50_ and average particle size *D*_av_ of the graded tailings were 33.01 μm and 50.59 μm, respectively. The graded tailings and cement have smooth particle gradation curves with curvature coefficients ranging from 1 to 3 and inhomogeneity coefficients > 5, which are all good gradations. The rod-ground sand has an inhomogeneity coefficient < 5 and is poorly graded [34]. Their chemical composition, mineral composition, and distribution were measured by X-ray fluorescence spectroscopy (XRF) (ZSX Primus II, Rigaku, Tokyo, Japan) and X-ray diffraction (XRD) (Ultima IV, Rigaku, Japan), and the results are shown in Table 1 and Figure 2. Of these, SiO_2_, Al_2_O_3_, and CaO have a relatively positive effect on coagulation and strength development [35].

**Figure 3:**
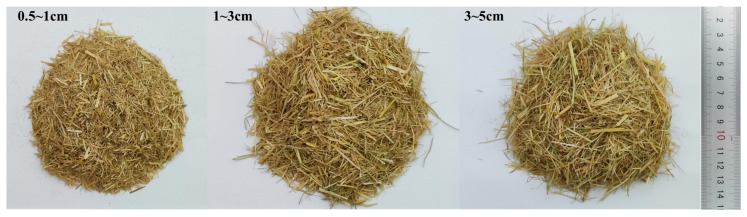
The morphology of rice straw fibers.

Rice straw fiber is mainly composed of cellulose (35.6%), hemicellulose (20.5%), lignin (16.8%), and ash (15.2%) [36]. Besides C, H, and O, the main element is Si. It has a low density and a high water absorption capacity, being able to absorb water equivalent to 300–517% of its own weight [15]. The tensile properties of rice straw fibers are weaker than those of other synthetic fibers such as polypropylene fibers, but still have a strength of 5.4 MPa and elongation of 2.3% [37]. Three lengths of rice straw fibers were used in this study: 0.5–1 cm, 1–3 cm, and 3–5 cm, as shown in Figure 3.

### 2.2. Specimen Preparation

There were 5 factors and 3 levels of RSCTB material proportion in this study. A total of 18 sets of test blocks were produced using the orthogonal test *L*_18_(3^5^). The orthogonal test was chosen because a complete test with multiple factors would have practical difficulties. For example, when considering 5 factors and 3 levels, the uniform design method requires 243 sets of tests, and the orthogonal design can greatly reduce the number of tests and improve the efficiency of the experiment.

The five factors were cement content in solids (*c*: 25.0, 20.0, and 16.7%, corresponding to cement–tailings ratio, respectively), solid mass concentration (*ω*: 73, 75, and 77 wt%), fiber content (*m*: 0.5, 1, and 1.5 kg·m^−3^), fiber length (*l:* 0.5~1, 1~3, and 3~5 cm) and rod-mill tailings addition (*r*: 0, 0.05, and 0.1 wt%). The proportions of the sets are shown in Table 2.

The raw materials were fully mixed in mortar mixer for 10 min and then stored in molds with dimensions of φ50 mm × 100 mm and φ50 mm × 50 mm. Then they were cured in a standard environment at 22 ± 1 °C and 90% relative humidity. A test block with the length-to-diameter ratio of 2 was used for unconfined static compressive strength, and the a block with the length-to-diameter ratio of 1 was used for the determination of dynamic compressive strength.

### 2.3. Experimental Methods

#### 2.3.1. Diffusivity Test

The diffusion cylinder was placed on a horizontally placed smooth steel plate, and the backfill slurry was poured through the upper opening of the diffusion cylinder. The diffusion cylinder was a cylindrical container with both the top and bottom diameters, as well as the height, all measuring 8 cm. After scraping the upper opening with a steel plate, the diffusion cylinder was quickly lifted vertically. The filling slurry diffused into a circle on the board. The diameter of this circle was measured in two directions, and their average value was used to characterize the diffusivity of the slurry.

#### 2.3.2. Unconfined Compressive Strength Test

UCS is an important characteristic to indicate the quality of the CTB. In this experiment, a computer-controlled automatic pressure testing machine WHY-200, produced by Shanghai Hualong Test Instruments Corporation (Shanghai, China), was used to test the specimens with different curing ages. The loading process was controlled by a computer with a loading rate of 0.2 mm/min. The strain rate during loading was 3.3 × 10^−5^ s^−1^.

#### 2.3.3. Dynamic Unconfined Compressive Strength Test

**Figure 4:**
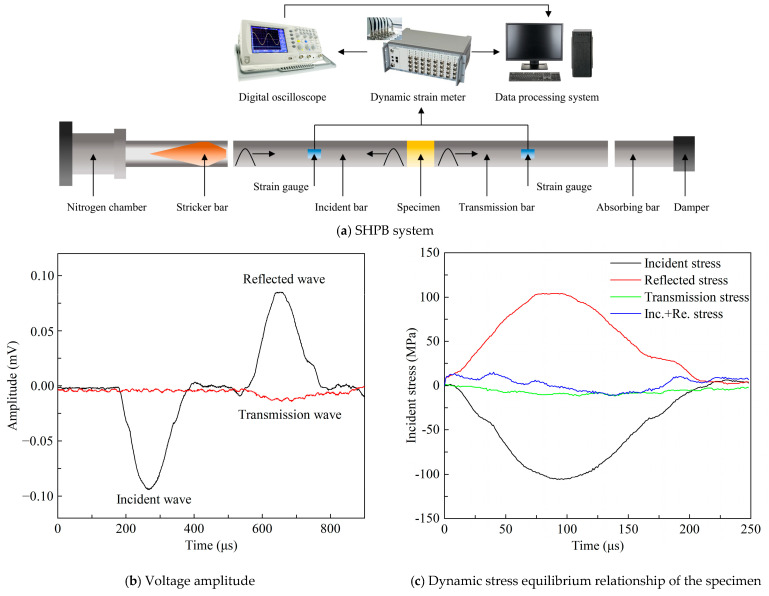
SHPB system and typical signal of tested specimens.

The strength performance of RSCTB under dynamic load was studied using SHPB (Central South University, Changsha, China). The SHPB can produce strain rates that are much greater than the UCS test. As shown in Figure 4, its experimental system consists of collision, incident, transmission, and absorption rods, which are all made of 40 Cr alloy steel with a diameter of 50 mm, a density of 7.81 g/cm^3^, an elastic wave speed of 5400 m/s, and a Young’s modulus of 240 GPa. The test dynamic loads were 0.4 MPa and 0.8 MPa, which were provided by the tanked nitrogen. Before the impact is loaded, the specimen end surfaces are polished and lubricated to ensure uniform stress distribution. The stress, strain, and strain rates were calculated by the following equations:

(1) $$\sigma (t)=\left(A_{B}E_{B}/2A_{S}\right)\left[\varepsilon_{i}(t)+\varepsilon_{r}(t)+\varepsilon_{t}(t)\right]$$

(2) $$\varepsilon (t)=\left(C_{e}/L_{S}\right)\int_{0}^{t}\left[\varepsilon_{i}(t)-\varepsilon_{r}(t)-\varepsilon_{t}(t)\right]dt$$

(3) $$\dot{\varepsilon }(t)=\left(C_{e}/L_{S}\right)\left[\varepsilon_{i}(t)-\varepsilon_{r}(t)-\varepsilon_{t}(t)\right]$$

where *σ*(*t*), *ε*(*t*), and $\dot{\varepsilon }$(*t*) are the stress, strain, and strain rate, respectively; *A*_B_ and *E*_B_ are the cross-sectional area and Young’s modulus of the steel rod, respectively; *A*_S_ and *L*_S_ are the cross-sectional area and length of the CTB, respectively; *C*_e_ is the wave velocity of the elastic wave in the rod; *t* is time; *ε*_i_(*t*), *ε*_r_(*t*), and *ε*_t_(*t*) are the signals of incident strain, reflected strain, and transmitted strain, respectively.

#### 2.3.4. Microstructure and Elemental Analysis

The Scanning Electron Microscope (SEM) (Quanta FEG 250, FEI Co., Hillsboro, OR, USA) was used to observe the microstructure of RSCTB. Energy-dispersive X-ray spectroscopy (EDS) was employed to measure the elemental content on the surface.

## 3. Results and Discussion

### 3.1. Fluidity

#### 3.1.1. Effect of RS Fiber on Backfill Slurry Fluidity

In this experiment, three groups of ratios were randomly selected in Table 2 as a blank control group without the addition of rice straw fibers, which were noted as T4/T7/T11 CTB, respectively. The raw material ratios and the condition of maintenance of the control group were the same as those of the normal group except that it did not contain rice straw fibers.

**Figure 5:**
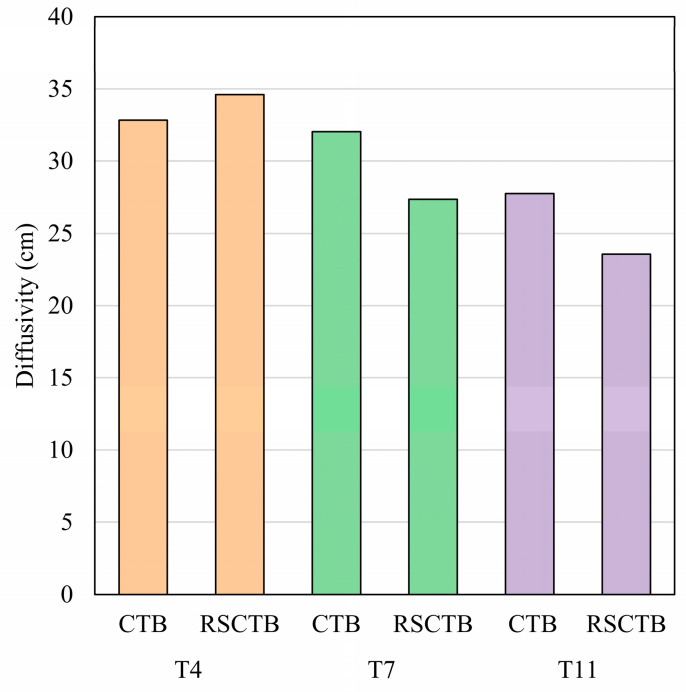
Diffusivity results of controlled experiments.

The results of the control experiments are presented in Figure 5. The diffusivity decreased by 15% in two of the three groups with added rice straw fibers and increased by 5% in the other group. Dried rice straw absorbs water during RSCTB preparation and impedes the flow of cement and tailing sand particles, both of which together contribute to the poorer fluidity of RSCTB compared to CTB without RS.

#### 3.1.2. Univariate Analysis of Factors Affecting RSCTB Slurry Fluidity

The results of the diffusivity experiments are shown in Table 3. Among the 18 sets, the mass concentration of the three groups of specimens with the smallest diffusivity were all 77%, which was the highest concentration in the experiment. The diffusivity of all other groups exceeded 19 cm, which could meet the backfill demand.

**Figure 6:**
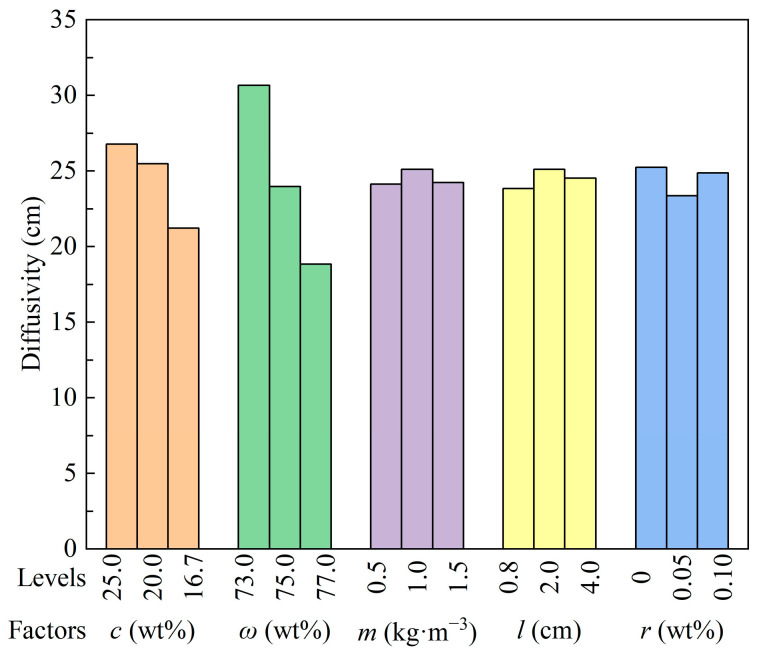
Average values of diffusivity at different levels of each factor.

The mean values of diffusivity for each factor at different levels are plotted in Figure 6. It can be seen that cement content is positively correlated with diffusivity, and that mass concentration and diffusivity are monotonically negatively correlated. The average particle size of tailings is larger than that of cement. As the ratio of cement to tailings decreases, the average particle size of the solids in the mixed mortar increases, which leads to an increase in the resistance in the flow. Contrary to the results of Chen et al. [15], the more fiber added or the longer the fiber, the poorer the flowability. The difference in the mean values of the results for different straw fiber additions and lengths in the present experiments was slight and not monotonically varying. This may be due to the different consistency of the prepared slurries and the different grain sizes of the tailings. The diffusivity is worst at 1.5 kg·m^−3^ fiber addition, fiber length 1~3 cm, respectively.

The mean value of diffusivity of RSCTB slurry without the addition of rod-mill tailings reached 25.25 cm, and the addition of a small amount of rod-mill tailings would weaken the mortar fluidity. In the preparation of straw fiber-reinforced CTB slurry, the appropriate fiber addition and fiber length should be selected according to the particle size composition of the solid particles and the flow performance requirements, to avoid adverse effects due to the reduction of slurry fluidity.

A higher value of Range (*R*_j_, j: *c*, *ω*, *m*, *l*, *r*) indicates that the corresponding factor has a greater influence on the diffusivity of the CTB. As shown in Table 4, the order of importance of factors affecting the diffusion of the CTB is Solid mass concentration (*ω*) > Cement content (*c*) > Rod-mill tailings content (*r*) > Fiber length (*l*) > Fiber content (*m*). Solid mass concentration is the most important factor affecting the fluidity. The length of the straw fibers selected in the experiments had a slightly higher effect on the flowability than the amount added, but much less than the solid mass concentration.

### 3.2. Unconfined Compressive Strength

#### 3.2.1. Enhancing Effect of RS Fiber on CTB

**Figure 7:**
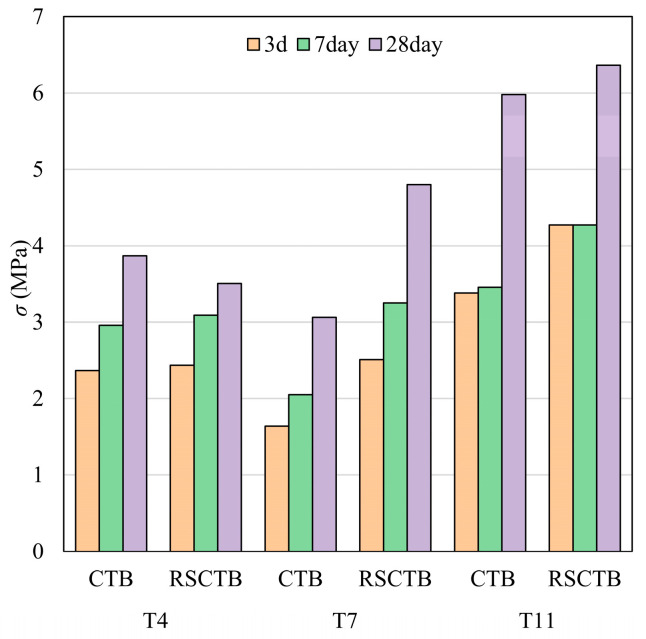
Unconfined compressive strength results of controlled experiments.

Previous studies by Chen [15] and Wang [38] have demonstrated that RS fibers strengthened CTB. Three sets of blank control specimens were also tested for UCS. The UCS is shown in Figure 7. Overall, the UCS of RSCTB was consistently higher than that of CTB at all curing ages, including at 3, 7, and 28 days. Among them, the strength improvement of T7 RSCTB was higher than 50% compared with CTB. This once again proved that RS has a positive effect on the strength of CTB.

#### 3.2.2. Univariate Analysis of Factors Affecting RSCTB UCS

Table 5 shows the average UCS (*σ*_s_) and static elastic moduli (*E*_s_) on the 3rd, 7th and 28th day obtained from tests. The lowest-average-strength specimen among different curing times is T16 (c = 16.7%, ω = 73%, m = 1.5 kg·m^−3^, l = 1~3 cm, r = 0.1%), which is because the cement content and solid mass concentration of T16 were the lowest among all specimens. When the backfill slurry is filled into the mined-out area, the sooner it solidifies and reaches a specific short-term or mid-term strength, the more beneficial it is for miners or machinery to operate on the top of backfill. The sooner it reaches a specific long-term strength, the more advantageous it is for excavating adjacent stopes. The shorter the mining–backfilling cycle, the higher the mining efficiency.

In 3 days, all samples reached a strength above 2 MPa, except for T16, and the highest reaching 4.14 MPa (T3). On the 7th day, the lowest strength was 2.63 MPa (T16 and T17), while the rest exceeded 3 MPa. For the 28-day strength, T4 and T14~17 were below 4 MPa, with specimen T3 having the highest strength, at 7.38 MPa. All specimens met the industrial strength standard of 2.5 MPa. To investigate the importance of the properties of straw fibers in the curing process, the data were analyzed based on the method for orthogonal range analysis. The results of the orthogonal analysis are listed in Table 6.

By comparing ranges of strength and elastic moduli at different levels for each factor variable, the contribution extent of each variable in different mortar solidification periods can be derived. During the curing process from 0 to 28 days, the cement–sand ratio was always the most influential factor affecting the strength of the CTB, followed by the solid mass concentration. The importance of fiber content, fiber length, and rod-mill tailings varied depending on the curing age. In the early stage of curing, the length and content of rice straw fibers had a greater effect on the UCS than the rod-mill tailings, while the opposite was true in the later stage.

The length of rice straw fibers had a greater influence on UCS than the content in the first 3 days, and the opposite was true for the elastic modulus. In the long run, the straw content had a greater influence than the length, both for UCS and *E*_s_. The length and content of rice straw fibers consistently had weaker effects on the elastic modulus than concentration and rod-mill tailings content, and cement content was less important than rice straw for the long-term elastic modulus.

**Figure 8:**
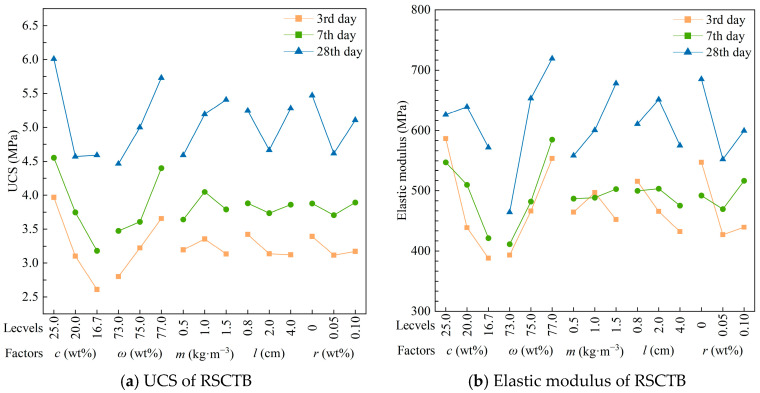
Single factor analysis of UCS and elastic modulus of RSCTB.

The compressive strength and elastic modulus of different levels of each factor are drawn in Figure 8. From Figure 8a, it can be seen that the UCS increases with the increase in curing time. The compressive strength decreases with decreasing cement–tailings ratio and increases with increasing mass concentration.

As the mass of added straw fibers increased, the UCS and *E*_s_ increased and then decreased on the 3rd day and continued to increase on the 28th day (Figure 8b). This phenomenon suggests that high fiber addition is detrimental to the early strength, which might be due to the insufficient hydration reaction in the first few days as a result of water absorption by the straw fiber. Meanwhile, the increase in fiber length also decreased *E*_s_. A small amount of rod-mill tailings has a significant inhibitory effect on UCS and *E*_s_, and the inhibitory effect is more obvious in the late maintenance period.

### 3.3. Compressive Response Under Dynamic Load

The results of the dynamic unconfined compressive strength (DUCS) *σ*_d0.4MPa_, strain rate ($\dot{\varepsilon }$), and dynamic increase factor of compressive strength (DIF*_σ_*) are shown in Table 7. The DIF*_σ_* is used to reflect the degree of change in strength under dynamic compression, which is calculated using Formula (4).

(4) $${\mathrm{DIF}}_{\sigma }=\frac{\sigma_{d}}{\sigma_{s}}$$

The dynamic compressive strength of the test specimens increased significantly with curing time. The average DUCS increased by 24.5%, from 7.59 MPa to 9.45 MPa. The average values of the strain rate at 0.4 MPa were 58.69 s^−1^ and 67.37 s^−1^ after the specimens had been cured for 3 and 28 days, and the compressive strength values ranged from 3.36 MPa to 14.22 MPa and 5.47 MPa to 21.88 MPa, respectively. For the same proportioned specimens, the DUCS was 1.34–3.12 times (3 days) and 1.39–3.33 times (28 days) of the UCS.

Taking specimen T6 as an example, its strength under static load with a loading rate of 2 × 10^−4^ s^−1^ was 3.88 MPa (3 days) and 6.52 MPa (28 days), and when the strain rate increased to more than 50 s^−1^, the compressive strength was 9.53 MPa (3 days) and 13.82 MPa (28 days), which are 2.46 times and 2.12 times that of the static compressive strength. Severe localized damage was produced at the ends of the tested specimens before the formation of a macro-crack penetrated entire specimens under the high loading rate and short force action time. The volumetric strain generated outward in the middle of the specimens was extremely limited, leaving it in an approximate confined compression. Specimens did not have enough time to deform, thus leading to a higher compressive strength than static loading, along with a lift in elastic modulus.

**Figure 9:**
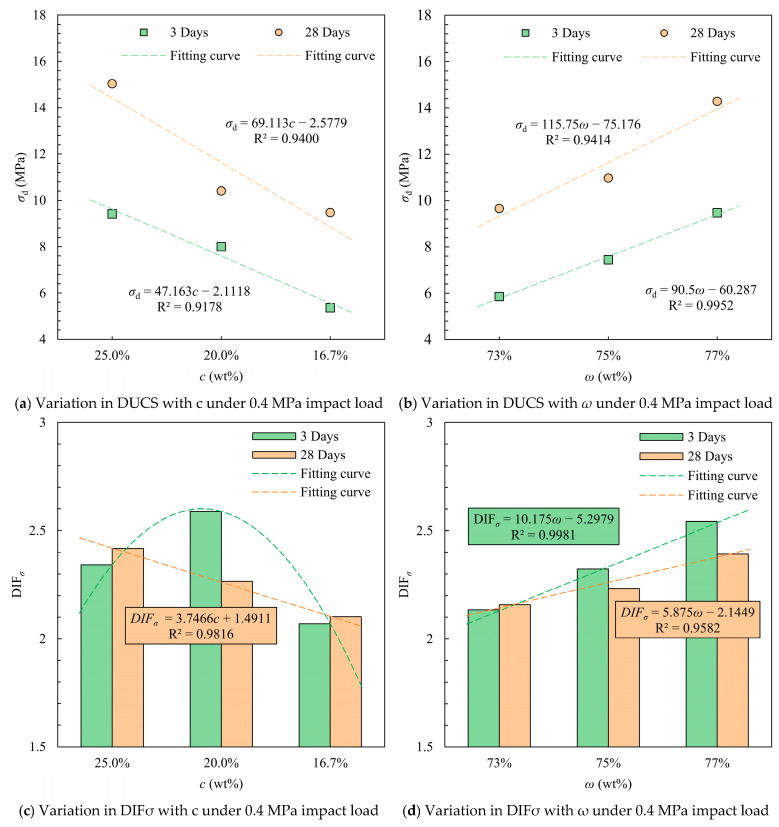
Mean values of DUCS and DIF*_σ_* for different *c* and *ω* conditions under impact load at 0.4 MPa.

DUCS showed a monotonically positive correlation with cement content and solid mass concentration in the 0.4 MPa dynamic load experiment. On the 3rd day, the average DUCS of the specimens with a cement–sand ratio of 1:4 was higher by 1.413 MPa (17.7%) and 3.873 MPa (72.3%) compared to the 1:5 and 1:6 specimens, respectively, and increased to 4.625 MPa (44.4%) and 5.565 MPa (58.8%) on the 28th day (Figure 9). A 4% higher solid mass concentration resulted in a 61.9% increase in initial strength and a 48.0% increase in long-term strength.

The DIF*_σ_* values also increased with increasing cement content and solid mass concentration. The 3-day DIF*_σ_* values were all greater than 2 and generally greater than the 28-day DIF*_σ_*, which indicates that the strength of CTB under dynamic stress is considerably higher than that under quasi-static loading, and also indicates that CTB, which does not have a high initial strength, has a greater increase in strength under the impact of a lower strain rate. As the gray-sand ratio becomes smaller, the DIF*_σ_* of 28 days decreases monotonically with the decrease. At curing time 3 d, the mean DUCS of 77% mass concentration was 2.027 MPa above 75% and 73% (27.2%), 3.62 MPa (61.9%) and 3.315 MPa (28.7%), 4.63 MPa (50.0%) at the 28th day. The DIF*_σ_* increases monotonically with increasing concentration, and the DIF*_σ_* at 3 days increases faster than the DIF*_σ_* at the 7th day.

**Figure 10:**
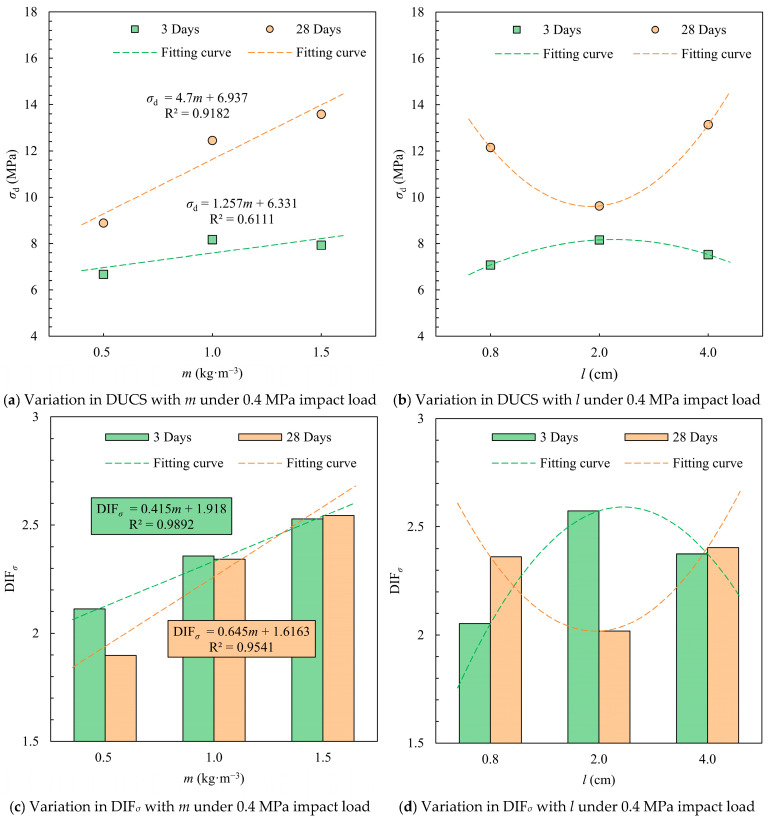
Mean values of DUCS and DIF*_σ_* for different *m* and *l* conditions under impact load at 0.4 MPa.

At 0.4 MPa impact, the highest dynamic compressive strength and dynamic increase factor (DIF) at 3 and 28 days were observed for the highest fiber content (1.5 kg·m^−3^). The average DUCS of the test specimens containing 1.5 kg·m^−3^ rice straw fibers was 7.93 MPa on the 3rd day, which was slightly lower than the average value of 8.17 MPa for 1 kg·m^−3^ RS fiber addition, but higher than the DUCS with the addition of 0.5 kg·m^−3^ (Figure 10). The dynamic enhancement factor increases with the amount of straw fiber added, and the more straw fiber added, the slower the rate of increase.

The average DUCS and DIF*_σ_* of specimens with added straw fiber lengths of 1–3 cm were the maximum among the three different lengths groups on day 3 and the minimum on day 28. This indicates that this specific fiber length has a stronger effect on the inchoate DUCS of the CTB, which has potential application in mines with frequent underground blasting disturbances. The fiber length to obtain the maximum DIF*_σ_* is not consistent for different maintenance times, and the appropriate fiber length can be selected when making backfill slurry based on the stoping cyclicality between rooms and pillars.

**Figure 11:**
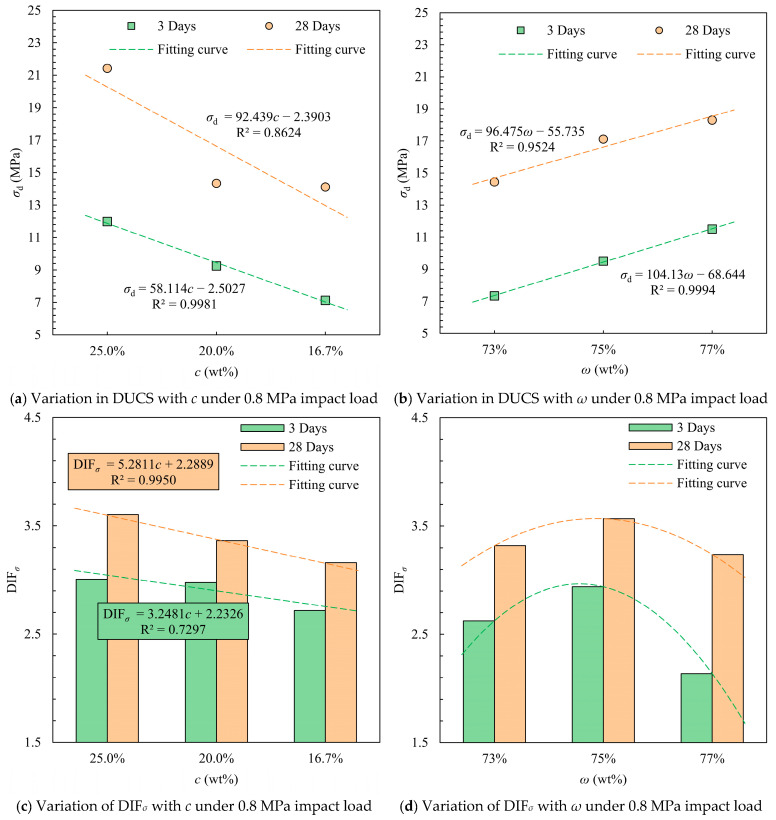
Mean values of DUCS and DIF*_σ_* for different *c* and *ω* conditions under impact load at 0.8 MPa.

The compressive performance of the specimens at 0.8 MPa was also obtained in this test. The test results are shown in Table 8. The average strain rate of CTB was 125.4 s^−1^ for 3 days curing time and 128.6 s^−1^ for 28 days, both about twice as high as that at 0.4 MPa. The values of DUCS were substantially higher after doubling the strain rate compared to those at lower strain rates, though the trends with *c* and *ω* did not change (Figure 11). The highest average DIF*_σ_* of 3 was recorded on day 3 of the specimens at *c* = 25%, while for the long-term strength, the lowest DIF*_σ_* value of 3.16 was recorded for the specimens with 16.7% cement content. The DIF*_σ_* values for long-term strength were significantly higher than those at the beginning of curing due to the increase in both strength and modulus of elasticity as the hydration reaction proceeded. The highest DIF*_σ_* was obtained for CTB with a mass concentration of 75% due to the fact that the specimens with a mass concentration of 77% obtained a higher baseline strength in the quasi-static pressure test despite having the highest DUCS.

**Figure 12:**
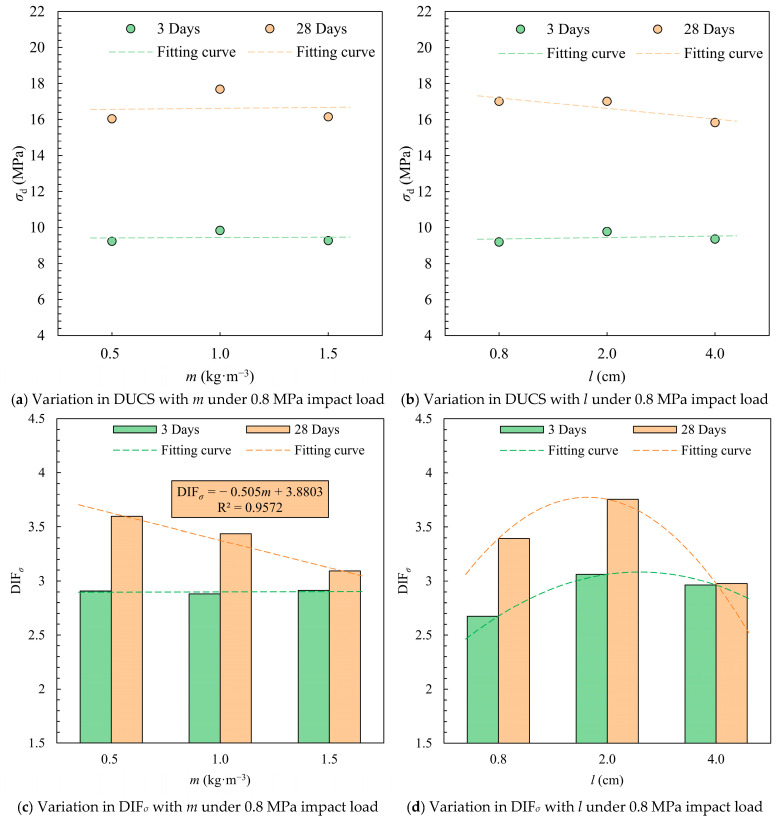
Mean values of DUCS and DIF*_σ_* for different *m* and *l* conditions under impact load at 0.8 MPa.

At 0.8 MPa impact, the maximum dynamic compressive strength occurred at a medium fiber content (1.0 kg·m^−3^). The specimens containing fibers of length 2.0 cm had the highest short- and long-term impact strength and optimal DIF*_σ_* (Figure 12). Different straw fiber contents at the beginning of consolidation had little effect on DIF*_σ_* at higher strain rates. Lower straw fiber content of 0.5 kg·m^−3^ resulted in higher DIF*_σ_* for long-term strength, but the DUCS was lower than the result of 1.0 kg·m^−3^, which was similar to the result of 1.5 kg·m^−3^. Although rice straw fibers can connect microfractures during the condensation process, too much rice straw intersects and overlaps in the CTB, making itself susceptible to becoming a weak link. The lowest 28-day DUCS mean value (15.84 MPa) was obtained for CTB with 4.0 cm straw fiber length, indicating that long fibers are not as good as short fibers in improving the impact resistance of CTB.

### 3.4. High Strain Rate Effect

**Figure 13:**
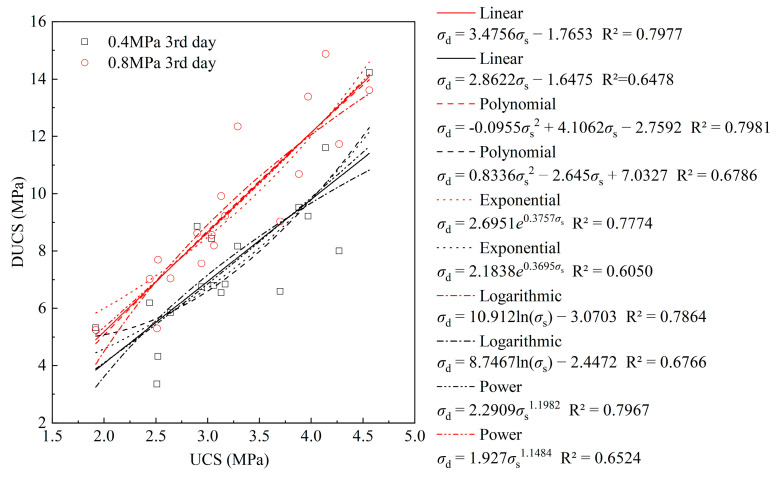
The strength curves fitted with *σ*_d_ and *σ*_s_ at different loading rates on the 3rd day.

Multiple functions were used to fit σ_s_ to σ_d_ in Figure 13. The correlation coefficient R^2^ shows that the quadratic polynomial has a high fitting accuracy when the strain rate is around 60 s^−1^. The power function has a high fitting accuracy when the strain rate is around 120 s^−1^. Overall, DUCS is positively correlated with UCS regardless of whether the impact strain rate is high or low.

Under higher air pressure, the impact device achieved higher initial acceleration and carried higher kinetic energy upon impact, resulting in greater deformation of the specimen per unit time and further reducing the generation of major fissure fractures in the middle of the specimen. The impact generated by the impact load is counteracted by the reinforcing stress only. Therefore, the specimens exhibited a relatively high strain rate under the impact load of 0.8 MPa, and also showed higher DUCS and DIF*_σ_*.

**Figure 14:**
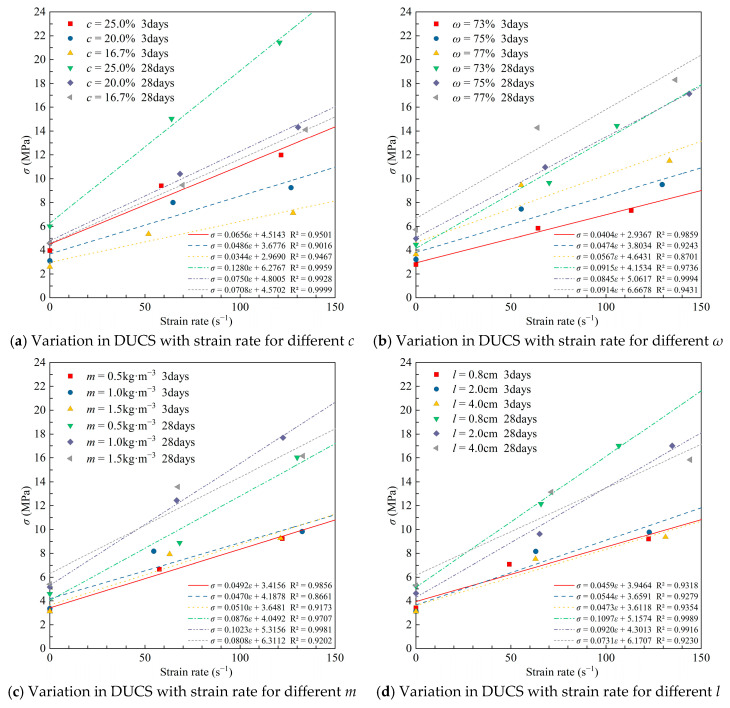
Fitting curves for DUCS and strain rate.

Regression analysis was performed on DUCS under different strain rates. The polynomial would over-fit the data to obtain an equation with R^2^ = 1, and the linear fit yielded the smallest R^2^ except polynomial (Figure 14). Some scholars have also found an exponential function as a better fitting relational model in similar studies, such as Cao and Chen.

Based on the fitting results, it can be seen that the rate of increase in DUCS with strain rate decreases when the cement–sand ratio decreases from 1:4 to 1:6 (Figure 14a). When the curing period lasted 28 days, the slope of the primary function of CTB with a cement content of 25% was much larger than that of other conditions, which indicated that the high binder content was beneficial to enhance the performance of CTB when resisting high load impacts. The strength enhancement at high strain rates was positively affected by the increased curing time.

Overall, the longer the curing time, the greater the slope of the fitted line. For higher strain rates, the RSCTB with 1.0 kg·m^−3^, 0.8 cm straw added achieved the maximum value of long-term strength for both factors, respectively. Therefore, in underground mining environments that are frequently subjected to blasting, the incorporation of 1.0 kg·m^−3^, 0.8 cm straw in CTB should be considered.

**Figure 15:**
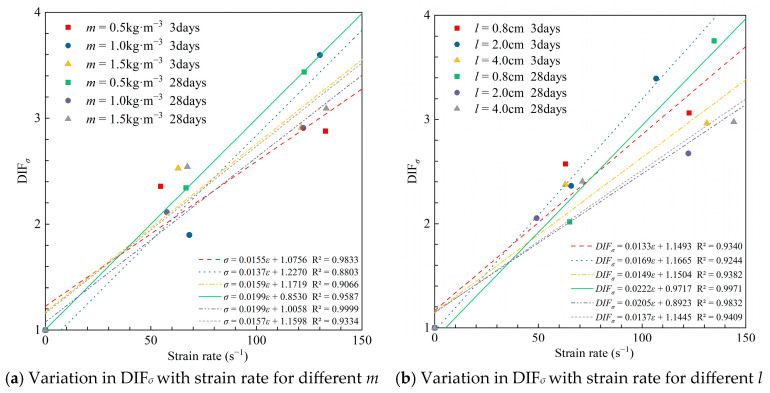
Fitting curves for DIF*_σ_* and strain rate.

Figure 15 shows the DIF*_σ_* of specimens with different rice straw fiber additions and fiber lengths at different strain rates. It can be seen that the DIF*_σ_* of specimens with higher fiber additions and longer fiber lengths in the late curing period was significantly lower than that of specimens with lower fiber content and shorter fiber lengths. This is another indication that a weaker structural surface was formed due to too many fibers or too-long fibers connected inside the specimens. The strength of the specimen is reduced because of the slip between the fibers when subjected to dynamic load. The suitable amount of suitable-length straw fibers added can give full play to their connection between the internal microcracks and weaken the negative impact on the strength.

### 3.5. Microscopic Morphology of RSCTB and Macroscopic Roles of RS Fibers

**Figure 16:**
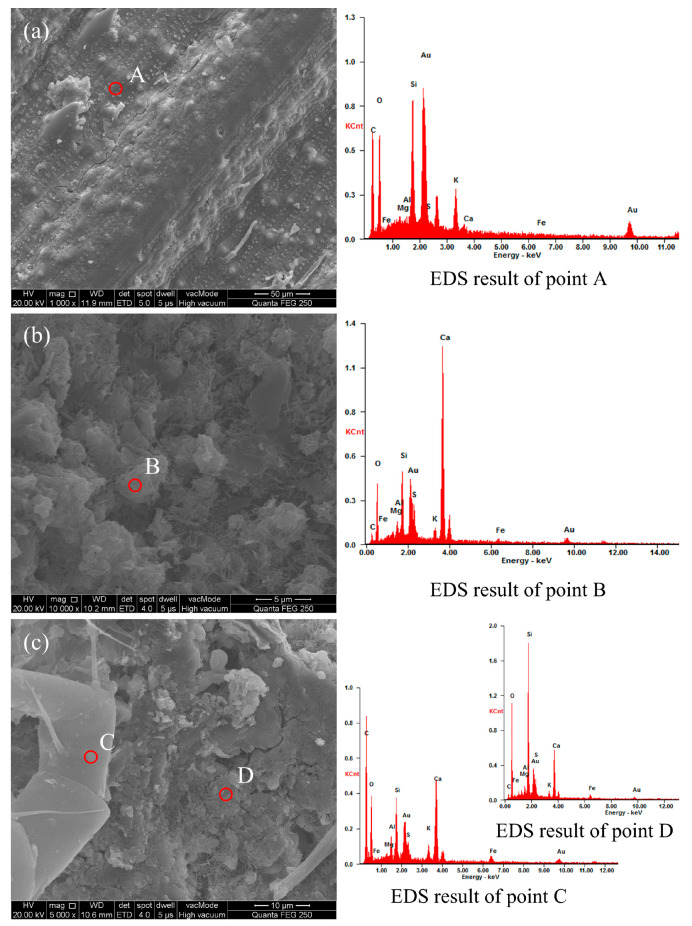
SEM and EDS results. (**a**) Rice straw; (**b**) RSCTB without RS fiber; (**c**) RSCTB with RS fiber.

Microscopic analysis of RSCTB helps to better understand the role of straw fibers during the consolidation of the backfill. Figure 16 presents the microstructural characteristics of straw fibers within RSCTB. Figure 16a shows the surface morphology of straw fibers. According to the EDS results in Table 9, measurement point A in the carbon element map has a mass fraction of 24.40%, primarily originating from organic components such as cellulose, hemicellulose, and lignin in the straw. A small amount of silicon (6.56%) comes from SiO_2_ absorbed by rice plants from the soil during growth. Longitudinal striations and protrusions are visible on the axial surface of the straw, and this rough surface texture facilitates the adhesion of cement hydration products.

The straw fibers are completely covered by CTB in Figure 16b. The EDS analysis at point B reveals that calcium is the dominant element, which is attributed to the formation of C-S-H gel through cement hydration—the primary contributor to RSCTB strength. In Figure 16c, the oxygen and carbon content at point C is significantly higher than at point D, which corresponds to hydration reaction products in the backfill. This indicates that point C represents micro-scale fiber tubes within RSCTB. These tubes can undergo flexible deformation under compression, which, on a macroscopic level, enhances the material’s toughness and impact resistance.

**Figure 17:**
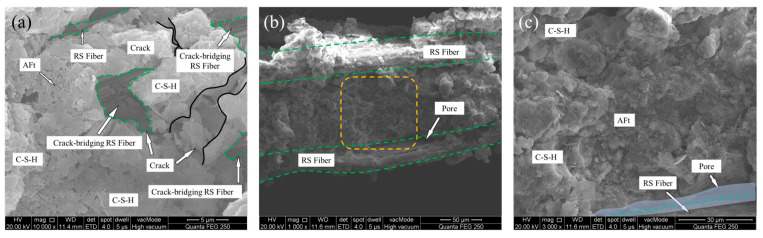
Microstructural features of RSCTB: (**a**) Rice straw fibers effectively bridging microcracks; (**b**) Relatively isolated rice straw fibers with almost no reinforcing effect; (**c**) Hydration products and the pores between them and rice straw fibers, shown as a local magnification of (**b**).

During the mixing process of the backfill slurry, the presence of air, as well as the consumption of water during cement hydration, inevitably leads to the formation of pores and microcracks within the hardened backfill. The microscopic cause of macroscopic failure in the backfill is the development of these microcracks, along with the connection and propagation of pores and fractures. Figure 17 illustrates the details of the interface between RS and CTB. In Figure 17a, RS fibers are encapsulated by C-S-H gel, with microcracks smaller than 5 μm present between the C-S-H structures. but the straw fibers bridge the solid blocks on either side of the pores well. When subjected to impact, the straw fibers act as a flexible skeleton, restricting the displacement of the solid material on both sides of the microcracks. This delays the development and propagation of microcracks and allows the fibers to maintain connectivity even after localized compressive failure, thereby enhancing the impact resistance of the backfill.

Figure 17b provides an overview of the bonding between RS fibers and the backfill, with the central region magnified in Figure 17c. The surfaces of the two RS fibers exhibit signs of cement hydration, with the presence of C-S-H attaching to the skeleton fibers. However, the pores between C-S-H and RS indicate that, while straw fibers improve the macroscopic strength of CTB, their effects at the microscopic level are not always beneficial. RS fibers that are not fully bonded with C-S-H may create weak structural interfaces within the material. Therefore, during the preparation of RSCTB slurry, thorough mixing is necessary to ensure adequate integration of RS with cement and to expel air.

**Figure 18:**
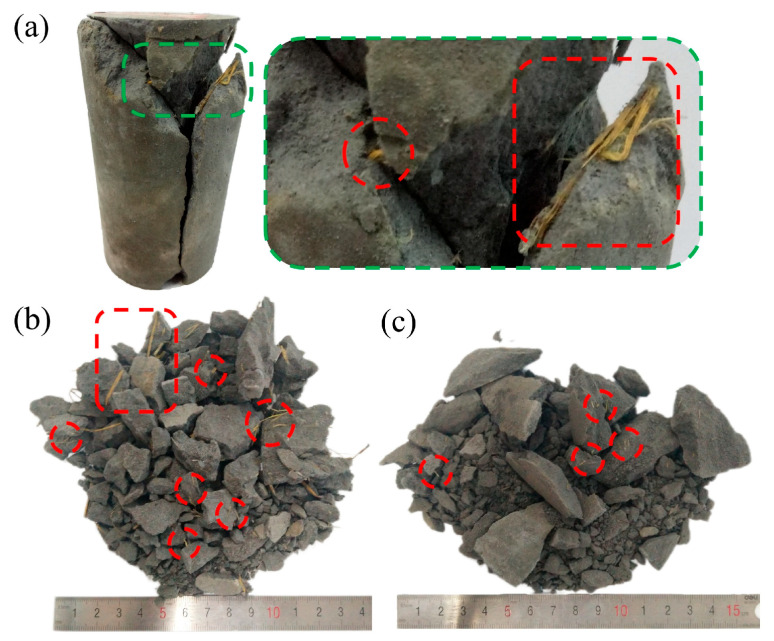
Post-failure appearances of RSCPB specimens. (**a**) Failure of RSCPB specimen after UCS test; (**b**) T3 RSCPB specimen after SHPB impact at 0.4 MPa air pressure; (**c**) T6 RSCPB specimen after SHPB impact at 0.4 MPa air pressure.

Straw fibers, serving as a flexible reinforcement material, assume a curved configuration after being incorporated and mixed into the backfill. During the failure process of the RSCTB, pore structures begin to interconnect and propagate. At the macroscopic level, the behavior of straw fibers can be categorized into the following types:

- Effective bridging, contributing to structural integrity: When intersecting the natural pores of the backfill at a relatively large angle, straw fibers are capable of bridging across pore walls; this behavior is shown in Figure 17a, effectively linking the cemented matrix on both sides and mitigating overall failure. The corresponding macroscopic post-failure state is highlighted by the red circles in Figure 18a.
- Partial bridging during failure, insufficient to prevent structural breakdown: As shown in red circles in Figure 18b,c, the fibers provided a degree of connectivity during deformation but ultimately failed to prevent the material from disintegrating. This can be attributed to two primary factors: either the interfacial bonding strength was insufficient, resulting in fiber pull-out, or the tensile capacity of the fibers was exceeded, causing rupture.
- No observable reinforcement effect: In certain cases, straw fibers did not contribute to the structural reinforcement of the backfill. Such occurrences are more likely when the fibers are excessively long or densely clustered in localized regions. RSCTB T3 and T6 had the same straw content, while T3 had a longer straw fiber length, and these longer straws even become the starting points of crack development, as illustrated by the areas marked with rectangles in Figure 18a,b. At the microscopic level, these fibers are relatively independent, as shown in Figure 17b.

The primary reinforcement mechanism of straw fibers in backfill is crack bridging. “Effective bridging” and “partial bridging” are distinguished only by the post-failure state of fibers and fragments, yet both provide resistance during failure. Although the ratio of effective to partial bridging varies with the applied impact load, the bridging effect consistently plays a dominant role. As shown in Section 3.2.1, specimens with straw fiber addition exhibit higher strength than those without, providing strong evidence that the positive effect of an appropriate fiber dosage outweighs its negative impact.

## 4. Conclusions

In order to investigate the mechanical properties of RSCTB under different strain rates, we investigated the compressive strength of CTB under quasi-static and dynamic loading using a pressure testing machine and a separated Hopkinson press bar, and reached the following conclusions:

- Orthogonal test analysis showed that the effects of straw addition and straw fiber length on UCS, DUCS, and fluidity were much smaller than those of binder content and solid mass concentration.
- The strength of RSCTB under quasi-static loading was generally higher than that of CTB without RS, with a maximum lead of more than 50%.
- The straw absorbed water and weakened the mobility of fresh CTB by up to 15%.
- High fiber content (1.5 kg·m^−3^) promotes the highest dynamic strength and DIF under low impact, while medium fiber content (1.0 kg·m^−3^) favors maximum strength under high impact.
- A moderate fiber length (1.0 cm) slightly reduced long-term strength under impact pressures below 0.4 MPa, but in all other cases, it was beneficial for dynamic compressive strength and the dynamic increase factor.
- The compressive properties of RSCTB are positively correlated with strain rate and can be fitted well by a linear function.
- Straw can bridge the pores within hydration products, providing flexible connections. However, excessive amounts or overly long fibers may adversely affect the performance of RSCTB.

Beyond mix proportioning, transportation tests are essential to assess the pumpability of fiber-reinforced slurry and to optimize its concentration and flow conditions. The incorporation of straw fibers requires systematic modifications to the current backfilling process, including fiber pretreatment (cutting, crushing, drying), precise dosing and feeding, and improved or prolonged mixing to ensure uniform dispersion. These process adjustments, aimed at balancing fiber reinforcement, slurry fluidity, and delivery stability, represent important directions for future research.

## Figures and Tables

**Table 1:** Main physical and chemical properties of cement, tailings, and rod-mill tailings.

Physical Property	Tailings	Rod-Mill Tailings	Cement
Bulk density *ρ* (t/m^3^)	1.93	1.86	
Particle density *ρ*_s_ (t/m^3^)	3.18	2.80	3.11
Porosity *n* (%) ^1^	39.31	33.57	
Osmotic coefficient *K* (cm/s)	8.70 × 10^−4^	3.80 × 10^−3^	
*D*_10_ (μm)	3.21	39.78	2.00
*D*_30_ (μm)	12.99	76.43	6.54
*D*_50_ (μm)	33.01	121.8	13.65
*D*_60_ (μm)	47.94	146.8	17.82
Uniformity coefficient *C*_u_^2^	14.93	3.69	8.90
Curvature coefficient *C*_c_^3^	1.10	1.00	1.20
Specific surface area *S* (m^2^/g)			1.25

^1^ *n* = (1 − *ρ*/*ρ*_s_) × 100%; ^2^ *C*_u_ = *D*_60_/*D*_10_; ^3^ *C*_c_ = (*D*_30_)^2^/(D_10_ × D_60_).

**Table 2:** Orthogonal table L18(3^5^) of RSCTB specimens.

Specimen Number	Cement Content *c* (wt%)	Solid Mass Concentration *ω* (wt%)	Fiber Content *m* (kg·m^−3^)	Fiber Length *l* (cm)	Rod-Mill Tailings *r* (wt%)
T1	25.0	73	0.5	0.8	0
T2	25.0	75	1.0	2.0	0.05
T3	25.0	77	1.5	4.0	0.10
T4	20.0	73	0.5	2.0	0.05
T5	20.0	75	1.0	4.0	0.10
T6	20.0	77	1.5	0.8	0
T7	16.7	73	1.0	0.8	0.10
T8	16.7	75	1.5	2.0	0
T9	16.7	77	0.5	4.0	0.05
T10	25.0	73	1.5	4.0	0.05
T11	25.0	75	0.5	0.8	0.10
T12	25.0	77	1.0	2.0	0
T13	20.0	73	1.0	4.0	0
T14	20.0	75	1.5	0.8	0.05
T15	20.0	77	0.5	2.0	0.10
T16	16.7	73	1.5	2.0	0.10
T17	16.7	75	0.5	4.0	0
T18	16.7	77	1.0	0.8	0.05

**Table 3:** Diffusivity test results of RSCTB slurry.

Number	Diffusion Diameter (cm)			Number	Diffusion Diameter (cm)		
	X Direction	Y Direction	Average Value		X Direction	Y Direction	Average Value
T1	35.00	34.00	34.50	T10	28.70	28.90	28.80
T2	23.80	26.20	25.00	T11	23.30	23.80	23.55
T3	23.00	24.70	23.85	T12	25.30	24.60	24.95
T4	33.80	35.40	34.60	T13	30.20	29.30	29.75
T5	28.50	29.60	29.05	T14	23.50	23.50	23.50
T6	19.30	19.80	19.55	T15	16.00	16.90	16.45
T7	27.00	27.70	27.35	T16	28.90	29.10	29.00
T8	21.60	19.80	20.70	T17	22.10	22.00	22.05
T9	13.30	13.90	13.60	T18	14.60	14.60	14.60

**Table 4:** Diffusivity at different levels of each factor.

Cement Content *c*	Average	Solid Mass Concentration *ω*	Average	Fiber Content *m*	Average	Fiber Length *l*	Average	Rod-Mill Tailings Content *r*	Average
25.0%	26.775	73%	30.667	0.5	24.125	0.8	23.842	0	25.250
20.0%	25.483	75%	23.975	1.0	25.117	2.0	25.117	0.05	23.350
16.7%	21.217	77%	18.833	1.5	24.233	4.0	24.517	0.10	24.875
Range *R_j_*	5.558		11.834		0.992		1.275		1.900

**Table 5:** Results of unconfined compressive strength and static elastic modulus for different curing times.

Number	Average UCS *σ*_s_ (MPa)			Average Static Elastic Modulus *E*_s_ (MPa)		
	3 Days	7 Days	28 Days	3 Days	7 Days	28 Days
T1	3.70	4.19	5.03	691.53	414.36	375.33
T2	3.97	4.48	4.68	586.42	551.95	750.34
T3	4.14	5.66	7.38	576.45	696.75	644.52
T4	2.44	3.09	3.51	268.05	360.89	420.56
T5	2.90	3.54	5.16	424.33	359.50	605.60
T6	3.88	4.14	6.52	618.58	560.53	1106.68
T7	2.51	3.25	4.80	355.55	410.07	531.19
T8	2.64	3.10	5.72	457.66	394.47	873.87
T9	2.94	3.66	5.01	471.85	427.96	696.44
T10	3.17	3.59	5.59	358.50	422.61	509.92
T11	4.27	4.27	6.36	551.98	559.37	714.01
T12	4.56	5.12	7.00	754.05	638.45	762.39
T13	3.06	4.09	4.59	421.39	494.40	522.44
T14	3.04	3.62	3.97	437.50	575.85	505.93
T15	3.29	4.00	3.68	462.97	707.93	674.23
T16	1.92	2.63	3.26	265.55	365.61	428.35
T17	2.52	2.63	3.96	340.33	451.42	471.28
T18	3.13	3.81	4.79	438.96	477.89	430.58

**Table 6:** Single-factor analysis of unconfined compressive strength and elastic modulus at 3rd day, 7th day, and 28th day.

Property	Curing Age	Level	*c*	*ω*	*m*	*l*	*r*	Significance
*σ_s_*	3 Days	*k*_1_	3.968	2.800	3.193	3.422	3.393	*c* > *ω* > *l* > *r* > *m*
		*k*_2_	3.102	3.223	3.355	3.137	3.115	
		*k*_3_	2.610	3.657	3.132	3.122	3.172	
		*R_j_*	1.358	0.857	0.223	0.300	0.278	
	7 Days	*k*_1_	4.552	3.473	3.640	3.880	3.878	*c* > *ω* > *m* > *r* > *l*
		*k*_2_	3.747	3.607	4.048	3.737	3.708	
		*k*_3_	3.180	4.398	3.790	3.862	3.892	
		*R_j_*	1.372	0.925	0.408	0.143	0.184	
	28 Days	*k*_1_	6.007	4.463	4.592	5.245	5.470	*c* > *ω* > *r* > *m* > *l*
		*k*_2_	4.572	4.975	5.170	4.642	4.592	
		*k*_3_	4.590	5.730	5.407	5.282	5.107	
		*R_j_*	1.435	1.267	0.815	0.640	0.878	
*E*_s_	3 Days	*k*_1_	586.49	393.42	464.45	515.68	547.25	*c* > *ω* > *r* > *l* > *m*
		*k*_2_	438.80	466.37	496.78	465.78	426.88	
		*k*_3_	388.31	553.81	452.37	432.14	439.47	
		*R_j_*	198.17	160.38	44.41	83.54	120.38	
	7 Days	*k*_1_	547.25	411.32	486.99	499.68	492.27	*ω* > *c* > *r* > *l* > *m*
		*k*_2_	509.85	482.09	488.71	503.21	469.52	
		*k*_3_	421.23	584.92	502.63	475.44	516.54	
		*R_j_*	126.01	173.59	15.65	27.78	47.01	
	28 Days	*k*_1_	626.08	464.63	558.64	610.62	685.33	*ω* > *r* > *m* > *l* > *c*
		*k*_2_	639.24	653.50	600.42	651.62	552.29	
		*k*_3_	571.95	719.14	678.21	575.03	599.65	
		*R_j_*	67.29	254.51	119.57	76.59	133.04	

**Table 7:** Test results under 0.4 MPa dynamic load.

Number	3 Days			28 Days		
	*σ*_d0.4MPa_	$\dot{\varepsilon }$	*DIF_σ_*	*σ*_d0.4MPa_	$\dot{\varepsilon }$	*DIF_σ_*
1	6.59	49.95	1.78	7.88	60.57	1.57
2	9.21	51.90	2.32	7.55	54.21	1.61
3	11.60	64.36	2.80	21.88	48.87	2.96
4	6.19	69.08	2.54	7.16	58.19	2.04
5	8.86	59.17	3.06	14.94	64.87	2.90
6	9.53	52.46	2.46	13.82	58.04	2.12
7	3.36	48.53	1.34	10.42	72.87	2.17
8	5.84	44.24	2.21	7.94	69.87	1.39
9	6.75	44.01	2.30	10.61	78.46	2.12
10	6.84	75.16	2.16	16.41	82.07	2.94
11	8.00	43.73	1.87	15.05	67.91	2.37
12	14.22	66.24	3.12	21.43	69.98	3.06
13	6.80	68.47	2.22	7.84	80.18	1.71
14	8.43	66.89	2.77	13.22	77.96	3.33
15	8.17	72.12	2.48	5.47	71.17	1.49
16	5.32	74.42	2.77	8.22	67.02	2.52
17	4.32	66.41	1.71	7.12	73.01	1.80
18	6.55	39.22	2.09	12.50	57.47	2.61

**Table 8:** Test results of the samples under 0.8 MPa dynamic load.

Number	3 Days			28 Days		
	*σ*_d0.8MPa_	$\dot{\varepsilon }$	DIF*_σ_*	*σ*_d0.8MPa_	$\dot{\varepsilon }$	DIF*_σ_*
1	9.03	84.55	2.44	22.83	34.07	4.54
2	13.39	117.79	3.37	21.60	122.61	4.62
3	14.88	132.47	3.59	30.63	140.02	4.15
4	7.03	114.94	2.88	14.12	142.82	4.02
5	8.62	131.94	2.97	12.56	131.37	2.43
6	10.69	116.11	2.76	9.96	87.02	1.53
7	5.30	130.78	2.11	11.23	48.64	2.34
8	7.04	142.03	2.67	15.17	139.02	2.65
9	7.57	147.06	2.57	8.92	149.96	1.78
10	9.23	116.75	2.91	10.85	146.50	1.94
11	11.73	138.35	2.75	18.68	147.86	2.94
12	13.61	139.21	2.98	23.95	133.23	3.42
13	8.19	140.35	2.68	15.70	129.95	3.42
14	8.56	127.98	2.82	18.33	153.45	4.62
15	12.35	129.48	3.75	15.30	138.20	4.16
16	5.25	92.57	2.73	11.93	132.46	3.66
17	7.70	118.87	3.06	16.38	167.43	4.14
18	9.92	135.99	3.17	21.05	169.69	4.39

**Table 9:** Elemental composition from EDS analysis.

Element	Point A (wt%)	Point B (wt%)	Point C (wt%)	Point D (wt%)
C	24.40	02.89	38.09	04.58
O	17.16	22.03	18.61	35.28
Mg	00.45	00.73	00.48	00.65
Al	00.12	01.55	01.65	01.23
Si	06.56	06.20	04.48	21.97
S	00.35	03.29	01.77	03.18
K	03.85	01.89	01.97	01.46
Ca	00.25	33.04	11.32	12.54
Fe	00.59	01.57	02.37	03.11

## Data Availability

The original contributions presented in this study are included in the article. Further inquiries can be directed to the corresponding author.

## Abbreviations

The following abbreviations are used in this manuscript:

RSCTB	Rice Straw Fiber-Reinforced Cemented Tailings Backfill
UCS	Unconfined Compressive Strength
DUCS	Dynamic Unconfined Compressive Strength
SHPB	Split Hopkinson Pressure Bar
CTB	Cemented Tailings Backfill
SEM	Scanning Electron Microscope
EDS	Energy-Dispersive X-ray Spectroscopy
DIF	Dynamic Increase Factor
